# High-frequency low-intensity semiconductor laser irradiation enhances osteogenic differentiation of human cementoblast lineage cells

**DOI:** 10.1007/s10103-024-04127-7

**Published:** 2024-07-06

**Authors:** Ayaka Nakatani, Ryo Kunimatsu, Shuzo Sakata, Yuji Tsuka, Mutsumi Miyauchi, Takashi Takata, Kotaro Tanimoto

**Affiliations:** 1 Department of Orthodontics and Craniofacial Development Biology, Graduate School of Biomedical & Health Sciences, Hiroshima University, Hiroshima, Japan; 2 Department of Oral and Maxillofacial Pathobiology, Graduate School of Biomedical & Health Sciences, Hiroshima University, Hiroshima, Japan; 3 Shunan University, Shunan City, Shunan Japan

**Keywords:** Dental cementum, Cell osteogenic differentiation, Near-infrared diode laser, Photobiomodulation

## Abstract

**Purpose:**

Laser irradiation activates a range of cellular processes in the periodontal components and promotes tissue repair. However, its effect on osteogenic differentiation of human cementoblast lineage cells remains unclear. This study aimed to examine the effects of high-frequency semiconductor laser irradiation on the osteogenic differentiation of human cementoblast lineage (HCEM) cells.

**Methods:**

HCEM cells were cultured to reach 80% confluence and irradiated with a gallium-aluminum-arsenide (Ga-Al-As) semiconductor laser with a pulse width of 200 ns and wavelength of 910 at a dose of 0–2.0 J/cm^2^. The outcomes were assessed by analyzing the mRNA levels of alkaline phosphatase (ALP), runt-related transcription factor 2 (RUNX2), and type I collagen (COLL1) using real-time polymerase chain reaction (PCR) analysis 24 h after laser irradiation. Cell mineralization was evaluated using ALP activity, calcium deposition, and Alizarin Red staining.

**Results:**

The laser-irradiated HCEM cells showed significantly enhanced gene expression levels of ALP, RUNX2, and COLL1 as well as ALP activity and calcium concentration in the culture medium compared with the non-irradiated cells. In addition, enhanced calcification deposits were confirmed in the laser-irradiated group compared with the non-irradiated group at 21 and 28 days after the induction of osteogenic differentiation.

**Conclusion:**

High-frequency semiconductor laser irradiation enhances the osteogenic differentiation potential of cultured HCEM cells, underscoring its potential utility for periodontal tissue regeneration.

## Introduction

Periodontal tissue consists of the gingiva, alveolar bone, periodontal ligament, and various components of the cementum. The periodontal ligament is a dense fibrous connective tissue located between the cementum and alveolar bone, whose homeostasis is maintained by the expression and function of various cells and intrinsic factors. On the contrary, the cementum is a thin, highly mineralized tissue that covers the root surface of the teeth [1]. The differentiation of cementoblasts and periodontal ligament cells into hard tissue-forming cells plays a crucial role in the repair and regeneration of the periodontium attributed to occlusal trauma or periodontal disease. Recent reports indicate that cementoblasts, unlike periodontal ligament cells, are highly expressed. More specifically, the expression of F-spondin, cementum-derived protein, cementum protein 1, cementum-derived attachment protein, protein-tyrosine phosphatase-like member-a, and miRNA (miR-383, miR-628-5p, and ets variant 1) highlight the fact that cementoblasts could possess unique properties in periodontal repair [2].

Laser is an abbreviation for light amplification by stimulated emission of radiation (LASER). It is a specific electromagnetic wave generated by the inducing radiation of substances in the excited state and has various characteristics according to its wavelength. Therefore, its effectiveness and widespread application in the medical field has been reported.

Photobiomodulation (PBM) or low-level light therapy (LLLT), which provides relatively low-power intravital irradiation with light-emitting diode (LED) or laser light at a specific wavelength of red or near-infrared (NIR) (600–1100 nm), has been shown to induce tissue regeneration, pain relief, and inflammatory modulatory effects [3, 4]. Recently, researchers commonly use the term PBM instead of low-level laser therapy [5, 6]. In addition, PBM has been reportedly used in a variety of dental treatments, oral surgery, oral related pain, and implant dentistry [3, 7, 8]. More specifically, PBM has been found effective in clinical studies as an adjunct to non-surgical periodontal pocket treatment to promote wound healing and relieve pain after periodontal surgical treatment [9, 10]. More recently, high-frequency NIR laser equipment with a high peak power and short pulse duration has been developed, allowing higher accessibility to deep tissues, photochemical activity, and a smaller chance of thermal injury to biological tissues [11]. Moreover, high-frequency semiconductor laser irradiation reduces pain and inflammatory cytokines after tooth extraction and relieves pain caused by bisphosphonate-related osteonecrosis of the jaw [12, 13]. High-frequency semiconductor laser irradiation has also been suggested to relieve pain during orthodontic treatment [14]. Additionally, in vitro studies have demonstrated that high-frequency semiconductor laser irradiation enhances the proliferation and migration of human gingival epithelial cells and mouse calvarial osteoblasts [11, 15]. Furthermore, animal models suggest that high-frequency, low-level semiconductor laser irradiation enhances wound healing in both the soft and hard tissues of tooth extraction sockets, while high-frequency NIR semiconductor laser irradiation leads to metabolic activation of periodontal tissues and increases tooth movement [16, 17]. In addition, high-frequency pulsed semiconductor laser irradiation exhibits biological effects and suppresses bone resorption in a mouse model of periodontitis [18]. Considered together, these findings indicate that semiconductor laser irradiation may affect the regulation of bone metabolism in periodontal cells. However, the efficacy of high-frequency semiconductor lasers for periodontal tissue metabolism in the maxillofacial region is yet to be elucidated.

Therefore, this study aimed to investigate the effects of high-frequency semiconductor laser irradiation on the osteogenic differentiation of the human cementoblast cell line (HCEM), focusing on cells of the cementum with peculiar properties among periodontal tissues.

## Materials and methods

### Cell culture

Human extracted tooth root surface cementum was immortalized using human telomerase reverse transcriptase after treatment using the sequential enzymatic treatment method devised by Kitagawa et al. [19]. by the experimentation Committee of the Graduate School of Biomedical Science, Hiroshima University. Kitagawa et al., established the HCEM cell line in the department of Oral and Maxillofacial Pathobiology, Graduate School of Biomedical & Health Sciences, Hiroshima University, and provided us the cell line. In the present study, HCEM cell lines 2 was used to conduct the study.

The HCEM cells were cultured in alpha-minimum essential medium (α-MEM) (Sigma Aldrich, St. Louis, MO, USA) containing 10% fetal bovine serum (FBS; Daiichi Chemical, Tokyo, Japan), 60 µg/mL kanamycin (Meiji Seika Pharma, Tokyo, Japan), 250 µg/mL amphotericin B (ICN Biomedicals Corp., Costa Mesa, CA, USA), and 50 U/mL penicillin (Meiji Seika Pharma) at 37℃ and under 5% CO_2_ at atmospheric pressure.

### Laser irradiation

A gallium-aluminum-arsenide (Ga-Al-As) semiconductor laser (Lumix 2, Fisioline s.r.l., Verduno, Italy; main wavelength: 910 nm, maximal power: 45 W, average power: 300 mW, pulse width: 200 ns, maximum pulse repetition rate: 50 kHz, pulse duration: 200 ns, secondary and guiding wavelengths: both 650 nm) was used in the experiment. At the time of laser irradiation, the medium volume of each culture dish was adjusted to the depth of 2 mm from the well floor, and a fixed stand was used so that the height of the irradiation orifice was at the well floor to the position where the guide light covers the bottom of the dish. The height of the orifice was 35 mm for 6-well plates, 25 mm for 12-well plates, and 15 mm for 24-well plates, and laser irradiation was performed to obtain 2.02 J/cm^2^ energetic quantity per time. The following presets were used: program 1, pulse rate: 30 kHz and overall duty cycle: 0.6%. A VEGA power meter (Ophir Optronics Solutions, Ltd., Jerusalem, Israel) was used to monitor the output. Table 1 lists the descriptions and specifications of the physical parameters of the laser, energy densities, and doses used.

**Table 1:** Description and specification of the physical parameters of the laser, energy, density, and dose used

Parameter	Value
Wavelength	910 nm
Operation mode	pulse
Pulse duration	200 ns
Frequency	30 kHz
Peak power density	90 W/cm^2^
Average power	0.3 w
Average power density	0.3 W/cm^2^
Peak power	45 w

### Quantitative real-time polymerase chain reaction (PCR) analysis

HCEM cells were seeded in 6-well plates (FALCON, Flanklin Lakes, NJ, USA) at a density of 1 × 10^5^ cells/well, and the medium was changed once every 2 days [20, 21]. After reaching 80% confluence, the FBS level in the culture medium was set to 0% in a stepwise fashion and laser irradiation at 2.0 J/cm^2^ was performed. Trizol Isolation Reagent (Thermo Fisher Scientific, Inc., Waltham, MA, USA) was used to recover RNA from the cellular layers 24 h after laser irradiation, and a RNeasy Mini Kit (Qiagen, Hulsterweg, Netherlands) was used to extract total RNA. RNA concentration was determined by measuring the sample absorbance at 260 nm using the NanoDrop One/Onec spectrophotometer (Thermo Fisher Scientific, Inc). ReverTra ace (Toyobo, Osaka, Japan) and Random primer (Toyobo) were used to synthesize cDNA from 1 µg total RNA. The mRNA expression levels of alkaline phosphatase (*ALP*), type I collagen (*COLL1*), and runt-related transcription factor (*RUNX2*) were analyzed using the obtained samples and specific primers (Table 2) and SYBR Green Real-time PCR Master Mix (Toyobo) by the LightCycler System (LightCycler 480 II [Roche Diagnostic, Basel, Switzerland]). The relative mRNA expression levels were analyzed using the ∆∆Ct method and normalized to those of beta-actin (*ACTB*).

**Table 2:** The polymerase chain reaction (PCR) primer sequences for alkaline phosphatase (ALP), runt-related transcription factor 2 (RUNX2), type 1 collagen (COLL1), and glyceraldehyde 3 phosphate dehydrogenase (GAPDH)

Gene	Sequence
GAPDH	forward: CCACTCCTCCACCTTTGA
	reverse: CACCACCCTCCTGTTGCTGTA
ALP	forward: ATGGTGGACTGCTCACAAC
	reverse: GACGTAGTTCTGCTCGTGGA
COLL1	forward: GATTCCCTGGACCTAAAGGTGC
	reverse: AGCCTCTCCATCTTTGCCAGCA
RUNX2	forward: CCCAGTATGAGAGTAGGTGTCC
	reverse: GGGTAAGACTGGTCATAGGACC

### Determination of ALP enzyme activity

The HCEM cells were seeded in 12-well plates (Falcon) at a density of 2 × 10⁴ cells/well containing a-MEM with 10% FBS, which was changed every 2 days [20, 21]. After reaching confluence, the medium was changed to osteogenic differentiation inducing medium (ODM), a-MEM containing 10% FBS supplemented with 0.05 mM ascorbic acid [Sigma Aldrich], 10 mM β-glycerophosphate [Sigma Aldrich], and 100 mM dexamethasone [Sigma Aldrich]). Laser irradiation at 2.0 J/cm^2^ was performed for every ODM change every 2 days. On the 7th day after osteogenic differentiation was initiated, the cells were washed twice with PBS before adding 0.1% Triton-X-100 and left at room temperature for 10 min before sample extraction. The resulting extract was used in pNPP phosphatase assay kits (Bioassay Systems, Heyward, CA, USA) to determine ALP activity according to the passage method. The absorbance was read at 405 nm using a MultiskanTM FC microplate reader (Thermo Fisher Scientific).

### Quantitative determination of the calcium concentration analysis

The HCEM cells were cultured in 12-well plates (Falcon) at a density of 4 × 10⁴ cells/well, and a-MEM containing 10% FBS was changed every 2 days [20, 21]. After reaching 80% confluence, the medium was changed to ODM to induce osteogenic differentiation. The ODM was laser-irradiated at 2.0 J/cm^2^ once every 2 days. The cells were washed with 10 mM Tris-HCl buffer (pH 7) on the 14th day of osteogenic differentiation and extracted with 10% formic acid at room temperature. The calcium concentration was determined using a calcium E-test Wako (Wako Pure Chemical Industries, Ltd., Osaka, Japan), a colorimetric assay based on the OCPC method, to measure the absorbance of chromogenic products at a wavelength of 620 nm using the Multiskan TMFC microplate reader (Thermo Fisher Scientific).

### Assessment of calcified deposits using alizarin red staining

The HCEM cells were cultured in 24-well plates (Falcon) at a density of 2 × 10⁴ cells/well [20, 21], and the medium was changed every 2 days. After reaching 80% confluence, the cells were transferred to the ODM, which was changed once every 2 days, and laser irradiation at 2.0 J/cm^2^ was performed during every change. The cells were washed with PBS 21 and 28 days following the onset of osteogenic differentiation and fixed with a 37% formaldehyde solution (Wako Pure Chemical Industries, Ltd.) diluted to 4% with purified water. Following this, the cells were treated with 1% Alizarin Red S stain. The stained cells were rinsed several times with UrtraPure ^TM^ DNase/RNase-Free Distilled water (Thermo Fisher Scientific) and dried overnight.

### Statistical analyses

All the data are presented as mean ± standard deviation. Comparisons between groups were performed using the Mann–Whitney U test. The level of significance was set at * *p* < 0.05 or ** *p* < 0.01.

## Results

### Effects of high-frequency semiconductor laser irradiation on the gene expression levels of ALP, COLL1, and RUNX2 in the HCEM cells

**Fig. 1:**
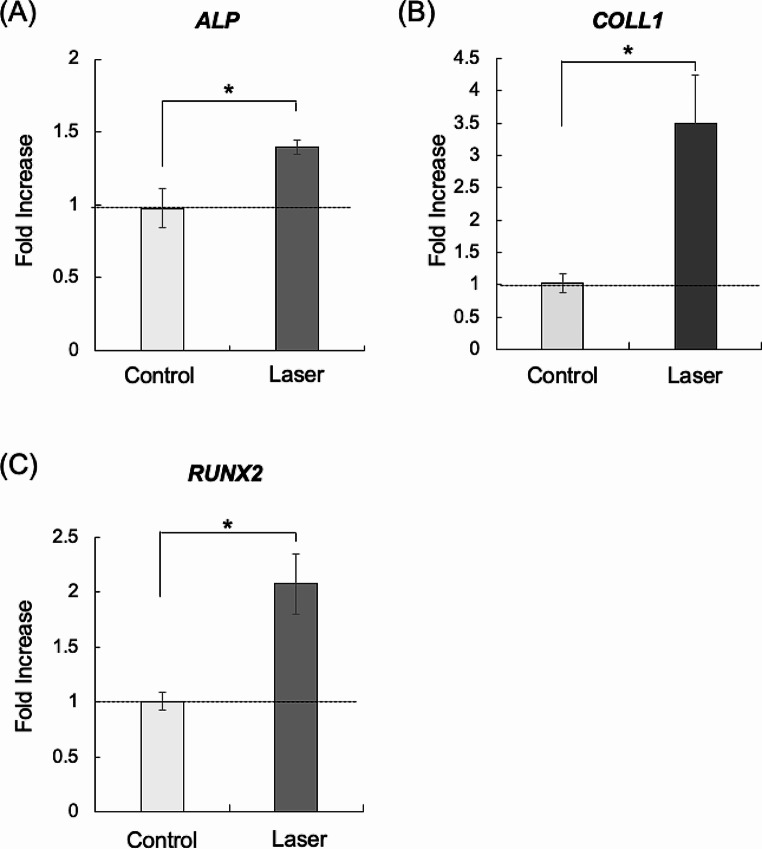
Effects of high-frequency semiconductor laser irradiation on gene expression levels of ALP, COLL1, and RUNX2 in HCEM cells. HCEM cells were cultured, and after reaching 80% confluence, FBS was shifted stepwise to 0% and the cells were laser irradiated. Gene expression levels of ALP, COLL1, and RUNX2 were assessed 24 h after laser irradiation. The mRNA expression levels of ALP, COLL1, and RUNX2 were significantly upregulated (*P* < 0.05) following high-frequency semiconductor laser irradiation (**A**, **B**, and **C**)

The expression levels of ALP, COLL1, and RUNX2 genes were examined 24 h after the HCEM cells were subjected to laser irradiation. There was a significant surge in ALP gene expression in the laser-irradiated group compared with the non-irradiated group (Fig. 1A). In addition, irradiation at 2 J/cm^2^ with a high-frequency semiconductor laser produced a near two-fold significant increase in the expression of RUNX2 and COLL1 genes compared with the non-irradiated group (Fig. 1B and C).

### Effects of high-frequency semiconductor laser irradiation on mineralization by the HCEM cells

**Fig. 2:**
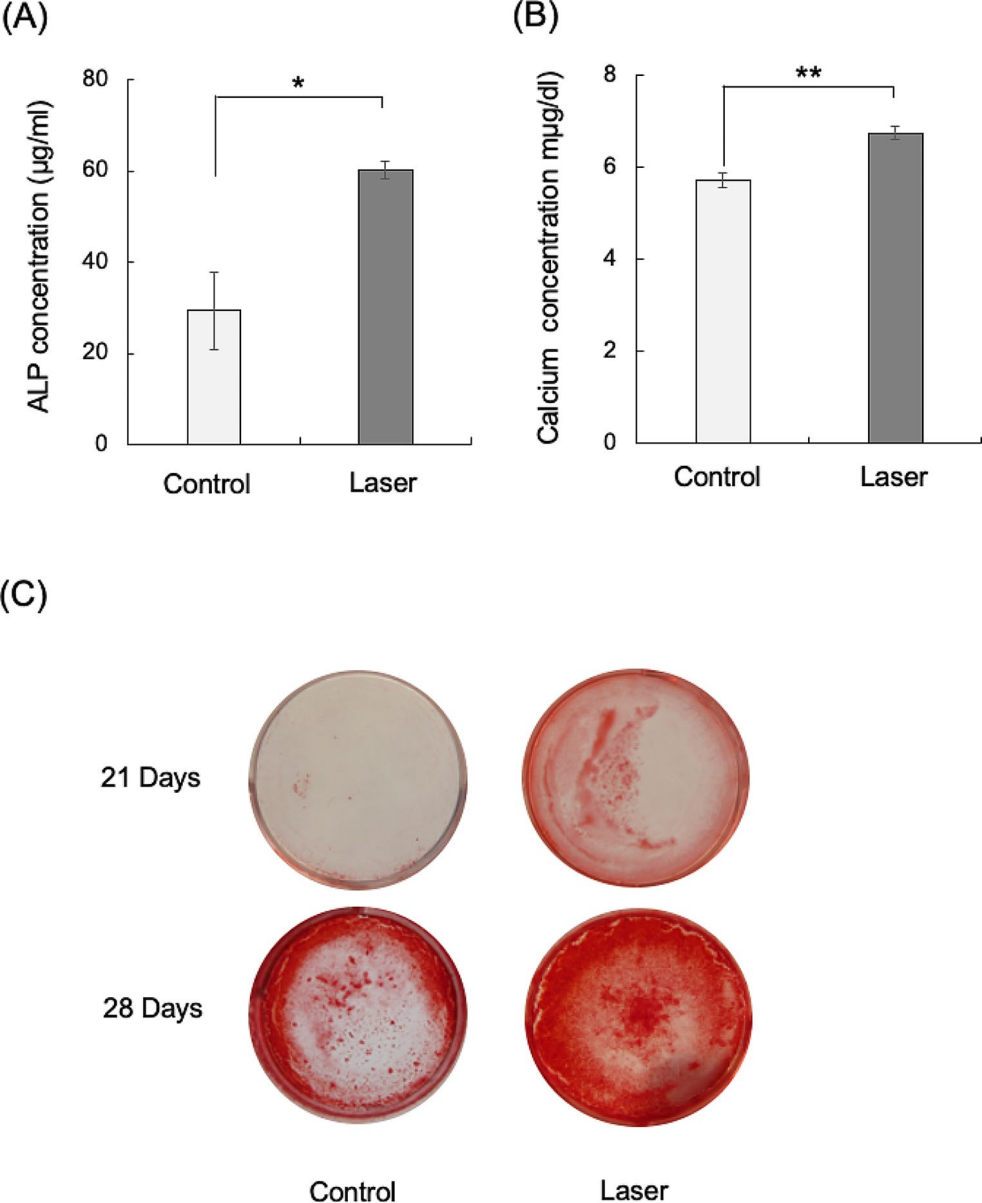
Effects of high-frequency semiconductor laser irradiation on mineralization by HCEM cells. HCEM cells were cultured and a-MEM containing 10% FBS and the medium was changed every 2 days. After reaching confluence, MEM containing 10% FBS was changed to osteogenic differentiation inducing medium (ODM). Laser irradiation was performed for every ODM change once every 2 days. (**A**) ALP activity: ALP activity in the HCEM cells treated with laser irradiation was significantly (*P* < 0.05) higher than that in the untreated control group 7 days after the induction of osteogenic differentiation. (**B**) Ca^2+^ concentration: The calcium concentration in HCEM cells was significantly enhanced (*P* < 0.05) by treatment with laser irradiation compared with that in the non-treated control groups 14 days after the induction of osteogenic differentiation. (**C**) Alizarin Red staining: HCEM cultures of the whole extracellular matrix region in ODM for 21 and 28 days were stained using Alizarin Red S. The staining level was also enhanced by laser irradiation compared with that in the untreated group

The ALP activity was significantly greater in the laser irradiated HCEM cells compared with the non-irradiated group 7 days after osteogenic differentiation was induced (Fig. 2A). In addition, the Ca concentration in the culture medium was significantly higher in the laser-irradiated group after 14 days of osteogenic differentiation (Fig. 2B). Finally, Alizarin Red staining showing calcified deposits was more noticeable in the laser-irradiated group on the 21st and 28th days of osteogenic differentiation (Fig. 2C).

## Discussion

The differentiation of osteogenesis-related cells involves the extracellular matrix and includes both collagen and non-collagen components [22]. In particular, ALP is known to be a key player in early-stage osteogenesis, while COLL1 production is considered an early marker of osteoblast differentiation [23, 24]. COLL1 is the main osteogenic process underlying the induction of ALP, and this phenomenon is thought to be the first step of cementoblastic differentiation [23, 25]. Moreover, RUNX2, an osteoblast-specific transcription factor expressed in differentiated osteoblasts and cementoblasts, has been implicated as a major regulator of osteoblast differentiation and gene expression [23, 26]. In addition, RUNX2 has been identified as a key factor in cementoblast and osteoblast differentiation [23]. Therefore, in the present study, we investigated the effects of laser irradiation on the gene expression profiles of ALP, RUNX2, and COLL1. We report a significant enhancement in the gene expression levels of ALP, RUNX2, and COLL1 in HCEM cells receiving 2.0 J/cm^2^ laser irradiation.

Laser irradiation with a semiconductor laser has been evaluated for effects on the osteogenesis-related gene expression. Studies have shown that visible semiconductor laser wavelengths of 600–700-nm irradiation promote bone differentiation in human-derived osteoblasts and enhance bone differentiation potential in bone marrow-derived mesenchymal stem cells (660 nm, 0.7–4 J/cm^2^) [8, 27, 28]. Additionally, semiconductor laser radiation (660 nm, 2–4 J/cm^2^) was found to enhance the expression of bone matrix protein (BMP)-2, osteocalcin (OCN), RUNX2, and ALP in periodontal ligament cells by semiconductor laser irradiation [29]. NIR semiconductor lasers with wavelengths of 700–900 nm have also been found to promote osteoblast (780 nm, 0.25–0.5 W) and bone marrow-derived mesenchymal stem cell osteogenic differentiation (810 nm, 2–6 J/cm^2^) [30, 31]. Interestingly, higher wavelengths (900–1000 nm) of semiconductor laser irradiation (940 nm, 1–1.5 W/cm^2^, and 3–4 J) were also found to significantly enhance gene expression of RUNX2, ALP, COLL1, BMP-2, Osterix, and TGFβ in MG-63 human osteoblast-like cells after 24 h [32]. While there are few reports on the effects of high-frequency semiconductor laser irradiation on osteogenic gene expression at a wavelength of 910 nm, its effects are consistent with previous reports.

In the present study, the effect of laser irradiation on the mineralization ability of human cementoblasts was changed to osteogenic differentiation induction medium after reaching confluence. The following effect was verified by examining the levels of ALP and calcium as well as Alizarin Red staining. Our results showed that laser-irradiated HCEM cells had significantly increased ALP concentrations compared with the non-irradiated group 7 days after the initiation of osteogenic differentiation, while the Ca^2+^ volume in the culture medium was significantly higher in the laser-irradiated group at the 14-day mark. In addition, enhancement of calcified deposits was observed in the laser-irradiated group at 21 and 28 days after bone differentiation was initiated.

To date, the effects of laser irradiation on osteogenic differentiation of cells have been investigated under various conditions. Previous studies have shown that ALP of human osteoblasts is increased by laser irradiation at 632 (0.43 J/cm^2^) and 660 nm (1.3 J/cm^2^) wavelengths [28, 33]. Wu et al. reported that 2- and 4-J/cm^2^ laser irradiation induced osteogenic differentiation of human periodontal ligament cells in a differentiation-inducing medium. Moreover, the study found that laser irradiation had enhanced the mineralization ability of human periodontal ligament cells at 660-nm wavelengths as assessed by osteogenic gene-expression, ALP quantitation, and Alizarin Red stain-based mineralization ability [29]. Additionally, light-emitting diode irradiation of differentiating mesenchymal stem cells with 8 J/cm^2^ at 660 nm has been shown to significantly increase RUNX2 gene expression, ALP activity, OCN protein concentration, procollagen type I C peptide-protein expression, Ca^2+^ concentration in culture broth, and calcification deposition assessed by Alizarin Red staining [34]. Additionally, a report on 800–1000-nm semiconductor laser irradiation showed that 905-nm (3.75-J/cm^2^) laser irradiation increased calcification of murine calvarial-derived osteoblasts [35]. Other studies reported that laser irradiation of human osteoblasts at 830 nm (3 J/cm^2^) increased ALP activity and highlighted an increase in BMP-2, BMP-4, and ALP gene expression as well as a significant increase in calcified deposits in human dental pump cells after 28 days of culturing following semiconductor laser irradiation at 810 nm (7.6 J/cm^2^) [36, 37].Regarding the effect of laser irradiation of 4 J/cm^2^ at 940 nm wavelengths on the osteogenic differentiation of inflamed periodontal ligament stem cells (I-PDLSC), Alizarin Red staining showed no significant changes after 14 and 21 days of induction of osteogenic differentiation. The ALP activity and expression of osteogenes​is-related​ genes reportedly increased significantly after laser irradiation [38].

To date, there has only been one report on the effects of laser irradiation on cementoblasts, in which semiconductor diode laser irradiation was performed at 940 nm (18 J/cm^2^) on mouse-derived cementoblasts (OCCM-30) seeded on root plates or microplates. The results showed a surge in the expression levels of BMP-2, 3, 6, 7; OCN; BSP; and Von Kossa staining, confirming enhanced mineralization [39]. However, the gene expression levels of RUNX2 and COLL1 did not differ significantly between the laser-irradiated and non-laser-irradiated groups [39]. It is also worth noting that the beneficial effects of laser irradiation on bone metabolism in cells, osteoblasts, and mesenchymal stem cells that comprise the periodontium have been discussed in several recent systematic reviews [18, 40–42]. Although the results of this study were similar to those of previous reports, there were differences in several endpoints. More specifically, the large variability of irradiation conditions, such as the type of laser, wavelength, irradiation time, energy quantity, and pulse setting between studies presents a significant barrier to the advancement of basic research on laser irradiation [18, 40–42].

The current study demonstrated that 2.0 J/cm^2^ laser irradiation at a wavelength of 910 nm may enhance the osteogenic differentiation of human cementoblast lineage cells. Nevertheless, its some key limitation has to be acknowledged. First, the laser used in this study was simultaneously irradiated by 650 nm of the guide beam as the sub-wavelength in addition to the dominant wavelength of 910 nm. Therefore, the outcomes of the current report should be interpreted with caution, and comparative examination of the effect of laser irradiation on cell metabolism at each wavelength should be performed in future studies with identical setting conditions. Second, regarding the signaling pathway of laser irradiation, previous basic studies have suggested laser irradiation to be involved in various signaling pathways. However, the signaling pathways and mechanisms associated with PBM have not yet been fully elucidated. Therefore, future studies should investigate the signaling pathway mechanisms of bone differentiation upon radiofrequency laser irradiation in cementoblasts and cells constituting periodontal tissues in more detail. Thirdly, the present study was an in vitro analysis, and the effect in vivo has not been elucidated. For clinical application, in vivo examinations should be carried out in the future, and further examination on the optimum condition of laser irradiation is warranted.

## Conclusion

This study revealed that high-frequency semiconductor laser irradiation determines a significant enhancement in the expression levels of osteogenesis-related genes ALP, RUNX2, and COLL1 in HCEM cells and increases the Ca^2+^ volume in the culture medium and number of calcified deposits, thus enhancing the mineralization ability of cultured human cementoblast lineage cells. This indicates its potential utility for periodontal tissue and cementum regeneration.

## Data Availability

Not applicable
